# Auditory cognition and perception of action video game players

**DOI:** 10.1038/s41598-020-71235-z

**Published:** 2020-09-01

**Authors:** Hannah J. Stewart, Jasmin L. Martinez, Audrey Perdew, C. Shawn Green, David R. Moore

**Affiliations:** 1 Communication Sciences Research Center, Cincinnati Children’s Hospital Medical Center, Cincinnati, OH 45229 USA; 2 Division of Psychology and Language Sciences, University College London, London, UK; 3 Department of Communication Sciences and Disorders, University of Cincinnati, Cincinnati, OH USA; 4 Department of Psychology, University of Wisconsin-Madison, Madison, WI USA; 5 Department of Otolaryngology, University of Cincinnati, Cincinnati, OH USA; 6 Manchester Centre for Audiology and Deafness, University of Manchester, Manchester, UK

**Keywords:** Psychology, Human behaviour

## Abstract

A training method to improve speech hearing in noise has proven elusive, with most methods failing to transfer to untrained tasks. One common approach to identify potentially viable training paradigms is to make use of cross-sectional designs. For instance, the consistent finding that people who chose to avidly engage with action video games as part of their normal life also show enhanced performance on non-game visual tasks has been used as a foundation to test the causal impact of such game play via true experiments (e.g., in more translational designs). However, little work has examined the association between action video game play and untrained auditory tasks, which would speak to the possible utility of using such games to improve speech hearing in noise. To examine this possibility, 80 participants with mixed action video game experience were tested on a visual reaction time task that has reliably shown superior performance in action video game players (AVGPs) compared to non-players (≤ 5 h/week across game categories) and multi-genre video game players (> 5 h/week across game categories). Auditory cognition and perception were tested using auditory reaction time and two speech-in-noise tasks. Performance of AVGPs on the visual task replicated previous positive findings. However, no significant benefit of action video game play was found on the auditory tasks. We suggest that, while AVGPs interact meaningfully with a rich visual environment during play, they may not interact with the games’ auditory environment. These results suggest that far transfer learning during action video game play is modality-specific and that an acoustically relevant auditory environment may be needed to improve auditory probabilistic thinking.

## Introduction

Computer-based sensory and cognitive training has long held the promise of dramatic improvements on real world abilities. However, finding an auditory training task that successfully leads to improved speech perception in noise, a frequently reported auditory disability^1,2^, remains elusive. Two problems have frequently occurred when training paradigms meant to improve such skills have been examined via carefully controlled experiments. The paradigms have either: (A) failed to produce benefits above and beyond those seen from placebo control conditions e.g., Ref.^3^; or (B) produced improvements on trained tasks, but with little improvement on untrained tasks, particularly those that were quite different from the trained task. Indeed, in a recent review of training studies aiming to improve auditory performance in adults with hearing loss, Ferguson and Henshaw^4^ clearly noted a general trend of improvement in the trained task (‘on-task’ learning) with little or no improvement in off-task abilities. Off-task abilities similar to those trained improved in some cases, a process termed ‘near transfer’, but there was little evidence of ‘far transfer’ to complex, off-task abilities. As an example of this latter situation, in one RCT^5^, training on a speech (phoneme) discrimination task^6^ produced robust on-task learning and some limited far transfer to auditory and visual divided attention and working memory tasks, but no generalized benefits for speech perception in noise.

In contrast to these often null or variable results, there has been a series of positive results demonstrating that training on one particular type of video game—dubbed action video games—produces enhanced, far transfer of visual cognition and visual perception abilities compared to non-players^7,8^. Action video games require the player to collect objects and avoid obstacles while battling enemies and maintaining their game character’s health and lives. The games are typically fast paced and require skills in hand to eye coordination and fast reaction times. Examples of such games are first and third person shooters—e.g., Call of Duty and Gears of War. A significant body of work in this domain has established that a causal relation exists between the act of playing action video games and the observed enhancements via controlled intervention studies (i.e., where individuals are specifically trained on either an action video game or a control video game e.g.,^9–12^). Yet much of the work in the field has been cross-sectional in nature e.g.,^13–15^. In such designs, the perceptual or cognitive skills of individuals who choose to play a great deal of action video games as part of their daily life (referred to as ‘action video game players’ or AVGPs) are contrasted against those of individuals who do not play such games (here labeled as ‘non-players’ or NPs). Although such cross-sectional designs cannot be used to infer a causal relation, the cross-sectional methodology has the advantage that because action video games are the most popular video game bought and played in America^16^, it is relatively easy to identify and recruit AVGPs. Given the extreme cost and difficulty of running full intervention studies, cross-sectional designs are thus often utilized by researchers to determine whether a full scale intervention is warranted (i.e., if AVGPs do not show enhanced performance on a given measure as compared to NPs, despite typically having played hundreds if not thousands of hours of action video games, it would seem unlikely that a training study where individuals are asked to play, at most tens of hours of action video games, would produce a significant effect).

One perceptual task that illustrates far transfer of training on action video games is the Multiple Object Tracking (MOT) task^17^. The basic MOT task consists of mentally labeling, continuously monitoring, then identifying the colour of up to 16 moving dots. Although the stimuli are far removed from popular ‘first person shooter’ action video games, the results nonetheless show that not only do AVGPs outperform NPs on this task^18–21^, but that deliberate action video game training also produces similar benefits, indicating that the relationship is causal^10^.

With AVGPs showing improvement on a wide range of skills assessed on tasks far removed from video game environments (for reviews see:^22,23^) it has been suggested that action gaming is training ‘probabilistic thinking’^24^. Auditory training paradigms have often attempted to train a restricted set of tasks and skills (e.g., speech phonemes^5^). This in turn often produces (at best) near transfer to outcome measures very similar or identical to the trained task. Instead, Bavelier et al. propose that action video game training induces a form of ‘learning to learn’ whereby individuals become generally better at learning to extract task relevant statistics. As a result, they are in turn better able to use a wide variety of task-relevant information occurring outside the trained game while ignoring distracting task-irrelevant information, as demonstrated by far transfer to complex tasks dissimilar to the trained task. Identification of a target in a noisy environment is a common challenge in sensory perception and pathology, for instance, attending to the relevant speaker while ignoring background speech. In terms of mechanisms, a test of the hypothesis of improved probabilistic thinking would be that the enhanced ability of AVGPs crosses modalities, evidenced by improved auditory cognition and perception.

There have been limited investigations into cross-modality improvements among AVGPs. Tetris, a visual puzzle game, has been found to improve frequency discrimination and auditory working memory when play was interspersed with a frequency discrimination task^25^. Interestingly, auditory cognition and perception did not improve when the participants were exposed to the tetris stimuli without the gaming environment. One AVGP study designed an auditory perceptual task to match a visual perceptual task^26^. Both tasks required a spatial decision about a target while the signal-to-noise ratio was manipulated. In the visual task the participants had to decide in which direction the majority of dots on a video display were moving for different levels of motion coherence. In the auditory task they had to decide in which ear they heard a target tone (the volume of which was adjusted between trials) while ignoring broadband noise in both ears. Both tasks showed that AVGPs were significantly faster than NPs at making these decisions, particularly at lower signal-to-noise ratios, while showing roughly equivalent levels of accuracy. This result suggests that AVGPs also have improved auditory cognition and perception, supporting cross-modal learning. However, the interpretation of this study is limited by the fact that the white noise masker would only have interfered with the target to the extent that it covers the same time/frequency regions. An informational masker, such as speech babble, would provide a more ecologically-valid masker requiring additional processes such as object formation and selection and linguistic processing^27,28^.

As in previous studies of video game players we used a self-report measure of the number of hours a range of gaming categories (e.g., first/third person shooter, turn-based strategy, music games, etc.) were played in the current year and previous years. Consistent with previous work demonstrating that such questionnaires only support the division of gamers into broad categories of play time^29^, we used four gaming classifications (full definitions in Table 1): AVGPs who played almost exclusively first/third person shooters; tweeners (TWs) who played multi-genre video games, typically online^30^; others (OTs) who do not fit a clear definition of gaming; and NPs who played at most 5 h a week across all game categories.

**Table 1:** Categorization rules: with four possible formula options for categorisation of action video game players (AVGPs) and one formula option each for categorisation of tweeners (TWs) and non-players (NPs) based on weekly hours of play during the past year, and prior to the past year. Participants that did not fall into these three categories were labelled as others (OTs).

	Action video game players (AVGPs)				Tweeners (TWs)	Non-players (NPs)
	Option 1	Option 2	Option 3	Option 4		
**Weekly hours of play during the past year**						
Action first/third person shooters e.g., Call of Duty, Gears of War	5 +	3 + to 5	3 + to 5	3 + to 5	–	0 to 1
Action RPG/sports/driving/adventure e.g., Mario Kart, Tomb Raider	–	–	5 +	5 +	0 to 10	0 to 1
Real-time strategy/MOBA e.g., Command and Conquer, Starcraft	0 to 3	0 to 3	0 to 3	0 to 3	0 to 10	0 to 1
Non-action turn-based role-playing/fantasy e.g., World of Warcraft, Pokemon	–	–	–	–	0 to 10	0 to 3
Turn-based strategy/life simulation/puzzle e.g., Sims, Candy Crush	0 to 3	0 to 3	0 to 3	0 to 3	0 to 10	0 to 3
Music games e.g., Guitar Hero, Rock Band	0 to 3	0 to 3	0 to 3	0 to 3	0 to 10	0 to 3
Other	0 to 3	0 to 3	0 to 3	0 to 3	0 to 10	0 to 3
Total hours played per week	–	–	–	–	–	0 to 5
**Weekly hours of play before the past year**						
Action first/third person shooters	–	5 +	3 + to 5	0 to 5	–	0 to 1
Action RPG/sports/driving/adventure	–	–	–	3 + to 5	0 to 10	0 to 1
Real-time strategy/MOBA	–	–	–	–	0 to 10	0 to 1
Non-action turn-based role-playing/fantasy	–	–	–	–	0 to 10	0 + to 3
Turn-based strategy/life simulation/puzzle	–	–	–	–	0 to 10	0 + to 3
Music games	–	–	–	–	0 to 10	0 + to 3
Other	–	–	–	–	0 to 10	0 + to 3
Total hours played per week	–	–	–	–	–	0 to 5

– = any amount of hours played.

This case–control study aimed to expand our understanding of the extent to which action video game experience is associated with cross-modal differences. We did this by examining performance on a variety of auditory tasks with varying demands, from simple RT based auditory attention to complex speech-in-babble identification. A finding of cross-modal differences in AVGPs as compared to NPs could prompt controlled intervention studies using action video games in therapeutic auditory training procedures. Thus moving away from training multiple specific mechanisms (whether auditory or cognitive) and towards the broader and potentially more motivating training offered by action video game play e.g.^31^. The participants were grouped by pre-existing gaming experience and received no study-related training. It was hypothesized that, compared to NPs, AVGPs would have better visual cognition and perception and better auditory cognition and perception. We expected that, across the tasks, TWs and OTs would perform numerically better than NPs but not as high as the genre-pure AVGPs.

## Results

**Figure 1:**
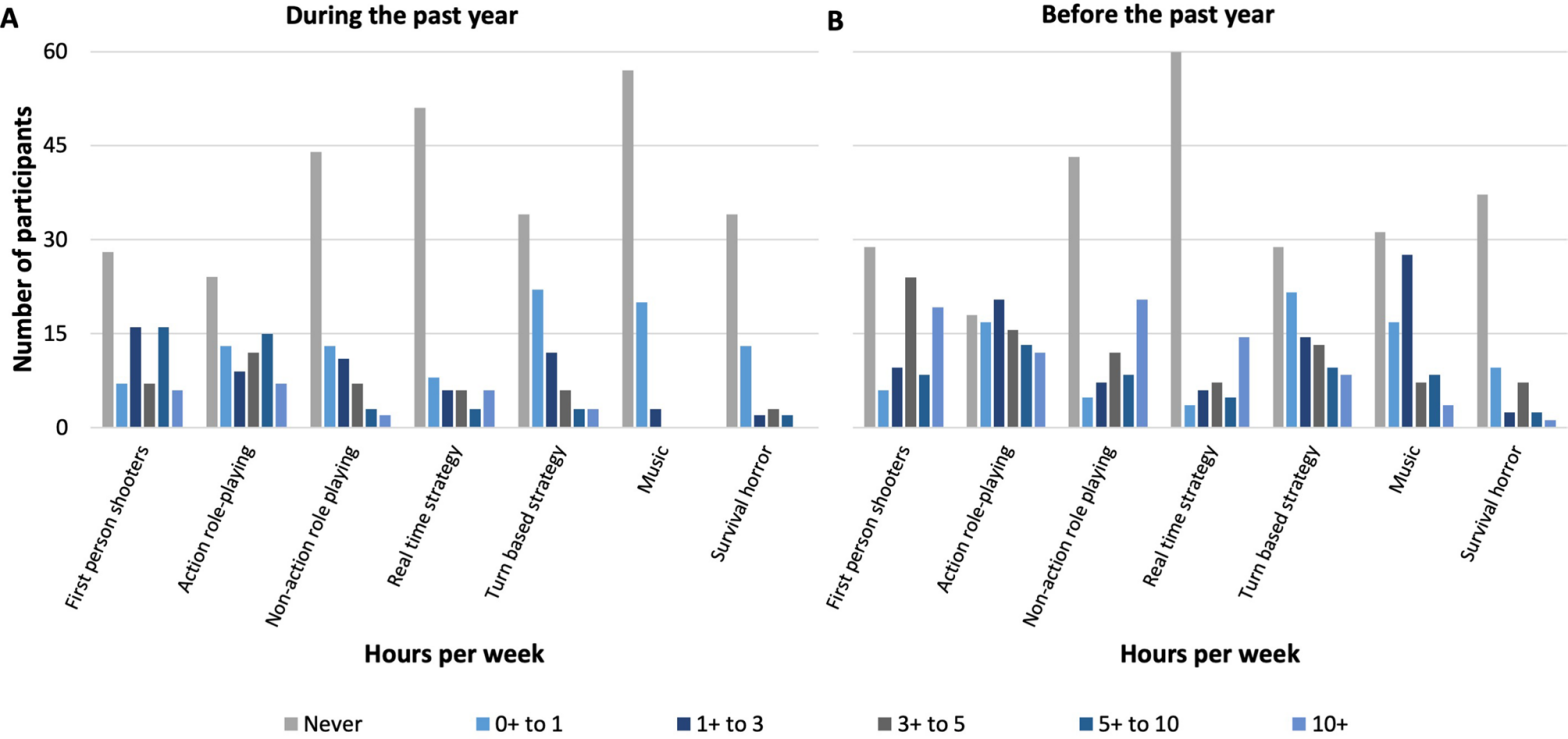
Self reported gaming experience across game categories played A. during the past year and B. before the past year. See Supplementary Material Fig. 1 for average hours per week.

We tested 80 participants (Table 2) with a range of gaming experience in the past year, and prior to the past year (see Fig. 1). Using strict categorization rules we separated the participants into AVGPs, TWs, OTs, and NPs (Table 1).

**Table 2:** Descriptives of participants tested including gender and age (*M *mean, *SD *standard deviation).

Group	n (F, M)	Age M (SD)
Action video game players (AVGPs)	15 (2, 13)	24.74 (3.11)
Tweeners (TWs)	17 (8, 9)	24.62 (2.88)
Others (OTs)	32 (3, 29)	25.52 (3.34)
Non-players (NPs)	16 (16, 0)	24.01 (2.40)

### Visual multiple object tracking (MOT)

**Figure 2:**
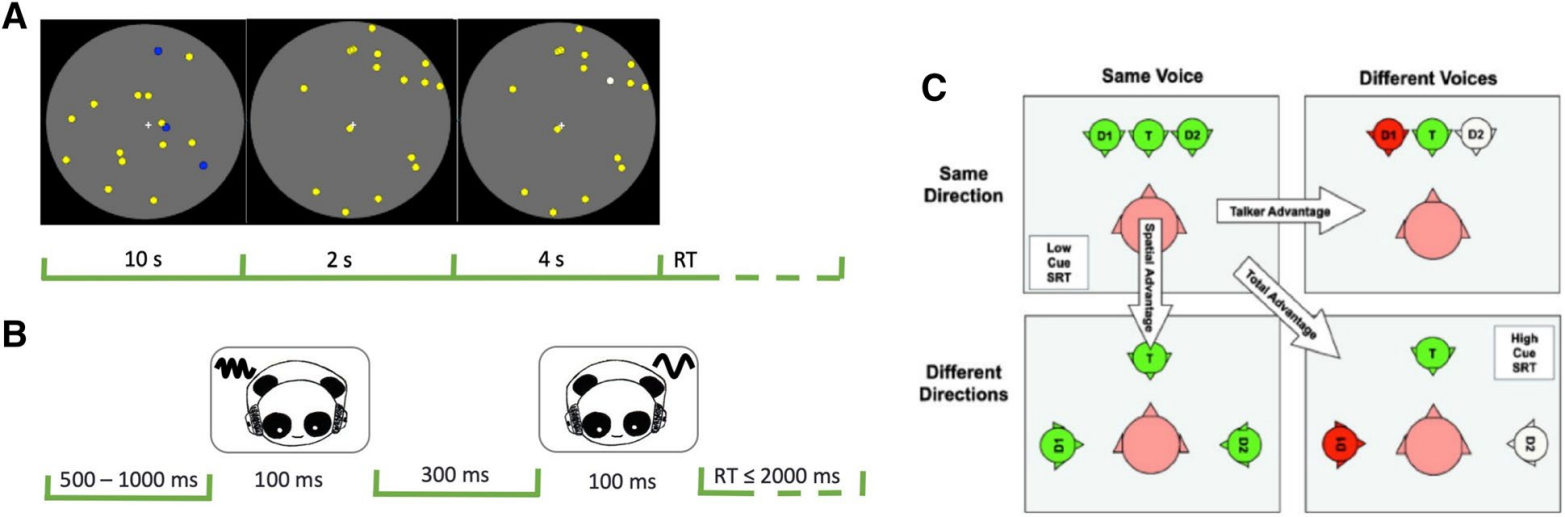
Paradigms for (**A**) Visual Multiple Object Tracking (MOT) task^17^, (**B**) Test of Attention in Listening (TAiL)^33^ and (**C**) Listening In Spatialized Noise—Sentences (LiSN-S)^36^. In a MOT trial the participants had to track the moving dots and at the end of the trial indicate via a button press whether the dot highlighted in white had started the trial as yellow or blue. In a TAiL trial participants heard two successive pure tones and had to indicate via a button press whether the frequency or location had changed or remained constant between the two pure tones. In the LiSN-S participants repeated the sentence of the target (T) while ignoring the adapting distractors (D1, D2) whose voices were manipulated to change their direction and/or voices (red and white heads) in four different condition blocks.

**Figure 3:**
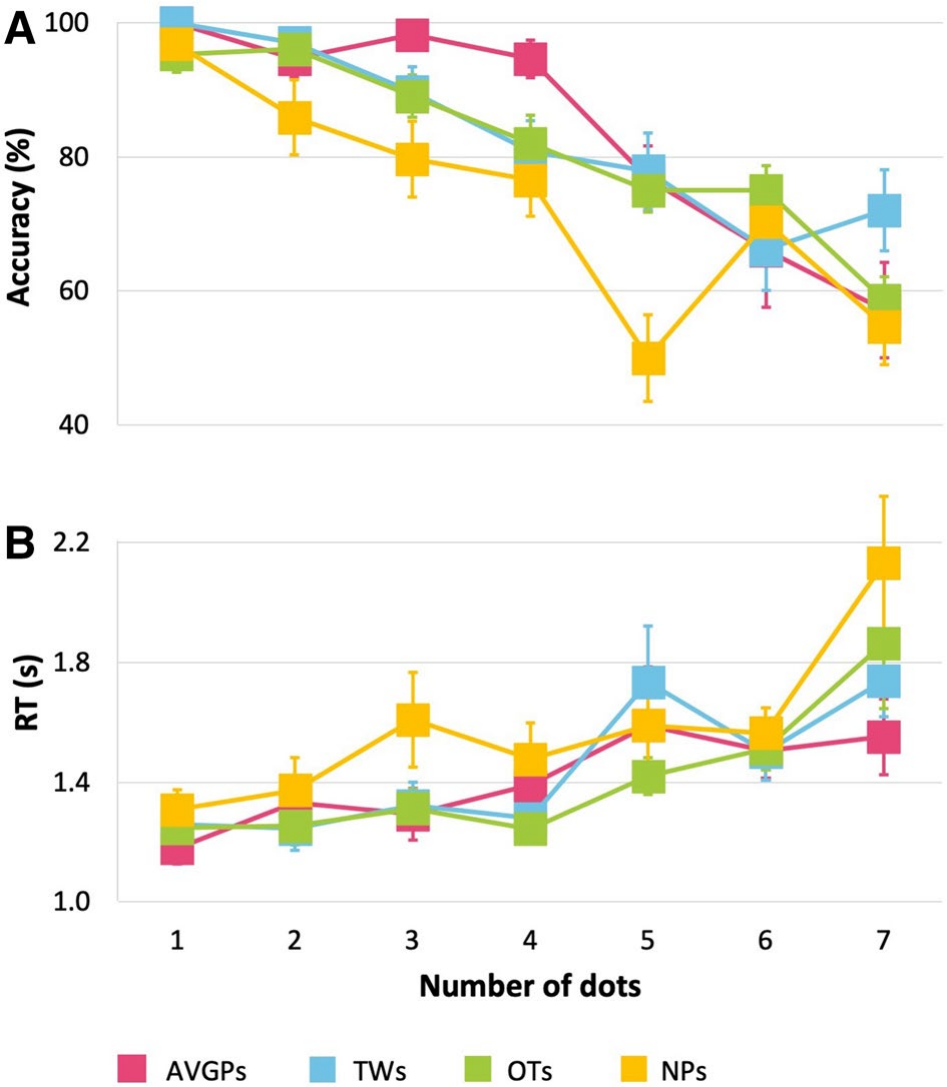
Multiple Object Tracking (MOT): Visual RT task (**A**) Accuracy (%) and (**B**) RT (s). Better performance is indicated by higher accuracy and lower RT. Pink: action video game players (AVGPs); blue: tweeners (TWs); green: others (OTs); yellow: non-players (NPs). Error bars show SEM.

We measured the ability of participants (n = 80; Tables 1, 2; Fig. 1) to perform the MOT (Fig. 2A). As noted previously, the MOT has previously been shown to demonstrate transfer of learning derived from action video game play. As expected, accuracy for detection of targets decreased and reaction time increased as the number of targets increased from 1–7 (Fig. 3).

Replicating previous work, AVGPs performed better than NPs (a history of at most 5 h a week experience across game categories). For accuracy, group (AVGPs, TWs, OTs and NPs) and number of blue dots (set size) were analyzed in a 4 × 7 repeated measures analysis of variance (ANOVA; Fig. 3A). Mauchly’s Test of Sphericity indicated that the assumption of sphericity had been violated, χ^2^(20) = 75.40, *p* < 0.001, therefore degrees of freedom were corrected using Greenhouse–Geisser estimates of sphericity. A main effect of group was observed, F(3, 75) = 5.79, *p* = 0.001, η_p_^2^ = 0.19, with AVGPs performing more accurately than the NPs (p = 0.004, η_p_^2^ = 0.40), as expected. TWs and OTs were more accurate than the NPs (*p* = 0.006, η_p_^2^ = − 0.37; *p* = 0.004, η_p_^2^ = − 0.40 respectively). A main effect of set size was also observed (F(4.69, 351.88) = 40.71, p < 0.001, η_p_^2^ = 0.35) with accuracy decreasing as the set size increased. Group interacted with set size, F(14.08, 351.88) = 1.94, *p* = 0.021, η_p_^2^ = 0.072). Post hoc *t* tests (all p-values are Bonferroni corrected) showed that this interaction was led by AVGPs performing more accurately than NPs at set size 3 (*p* = 0.025) and 5 (*p* = 0.005) and NPs less accurately than OTs and TWs at set size 7 (both *p* = 0.001).

The same design of ANOVA was run on reaction time (RT; Fig. 3B). Mauchly’s Test of Sphericity indicated that the assumption of sphericity had been violated, χ^2^(20) = 232.81, *p* < 0.001, therefore degrees of freedom were corrected using Greenhouse–Geisser estimates of sphericity. There was a main effect of set size, F(2.46, 174.53) = 13.30, *p* < 0.001, η_p_^2^ = 0.16, with slower reaction times for larger set sizes. No main effect of group was observed (F = 0.80, η_p_^2^ = 0.033, BF_10_ = 0.90) and no group interaction with set size (F = 1.05, η_p_^2^ = 0.042, BF_10MainEffects_/BF_10Interaction_ > 100).

### Test of Attention in Listening (TAiL)

**Figure 4:**
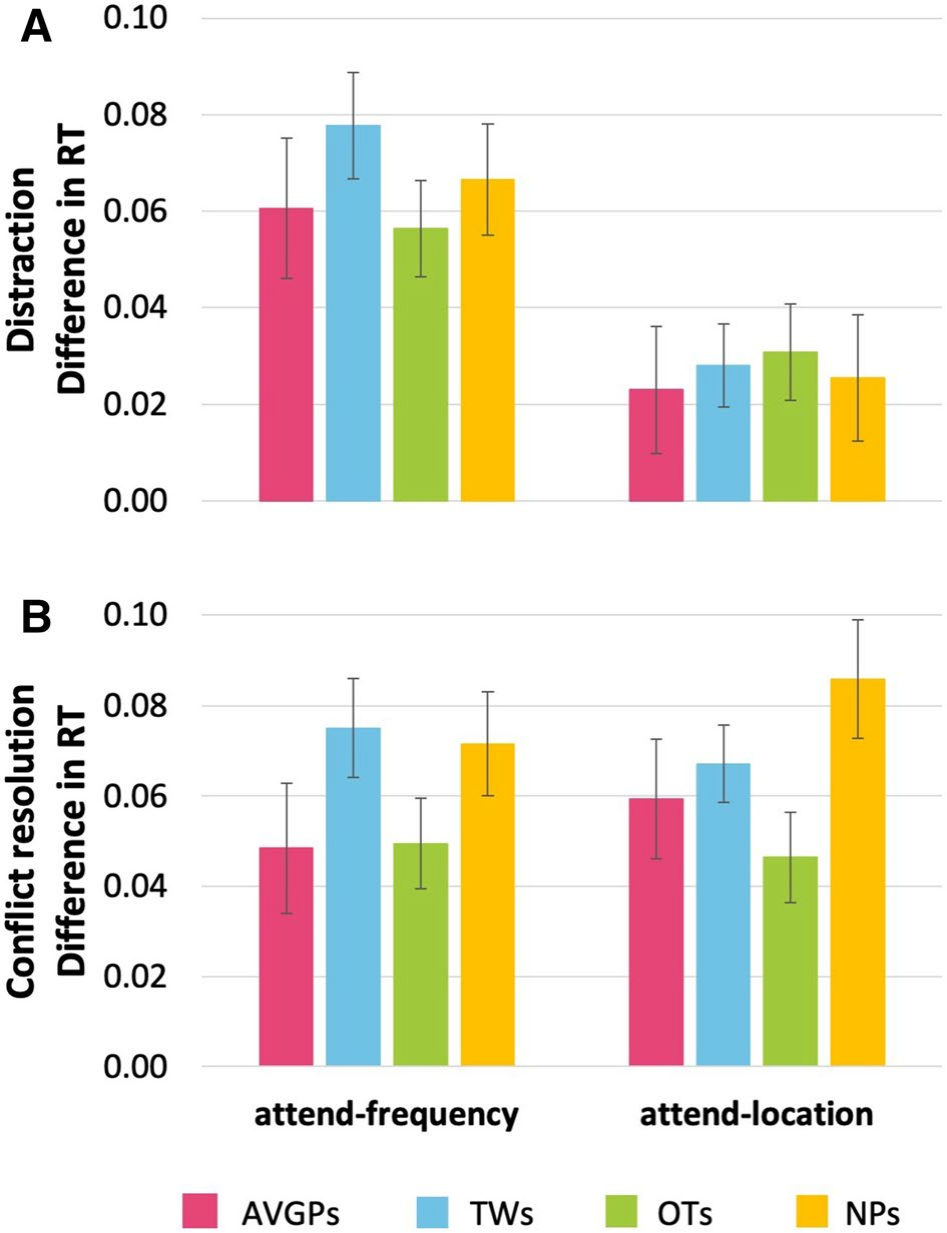
Test of Attention in Listening (TAiL): Auditory RT task (**A**) distraction (difference in RT) and (**B**) conflict resolution (difference in RT). A larger difference in RT for distraction indicates better performance as it reflects the ability to process task-irrelevant as well as task-relevant information. A smaller difference in RT for conflict resolution indicates better performance as it reflects that the participant is able to process incongruent information as well as congruent information. Pink: action video game players (AVGPs); blue: tweeners (TWs); green: others (OTs); yellow: non-players (NPs). Error bars show SEM.

TAiL (Fig. 2B) was developed as a simple, quick, multifaceted, RT-based index of attention modulation of auditory perception^32,33^. It measures the ability to focus on a task dimension, tone frequency or location, and ignore an irrelevant (distracting) dimension. Here, AVGPs and NPs were similarly distracted by task-irrelevant auditory information and their ability to deal with conflicting auditory information (Fig. 4). Univariate ANOVAs showed no group differences in distraction (attend-frequency: *p* = 0.58, η_p_^2^ = 0.026, BF_10_ = 0.15; attend-location: *p* = 0.93, η_p_^2^ = 0.006, BF_10_ = 0.087) or attend-frequency conflict resolution (*p* = 0.23, η_p_^2^ = 0.056, BF_10_ = 0.34). A group difference was found for attend-location conflict resolution (F(3, 75) = 2.94, *p* = 0.038, η_p_^2^ = 0.10). This was led by NPs being significantly more conflicted than OTs (*p* = 0.029, η_p_^2^ = 0.75).

### Bamford–Kowal–Bench Speech-in-Noise (BKB-SiN)

**Figure 5:**
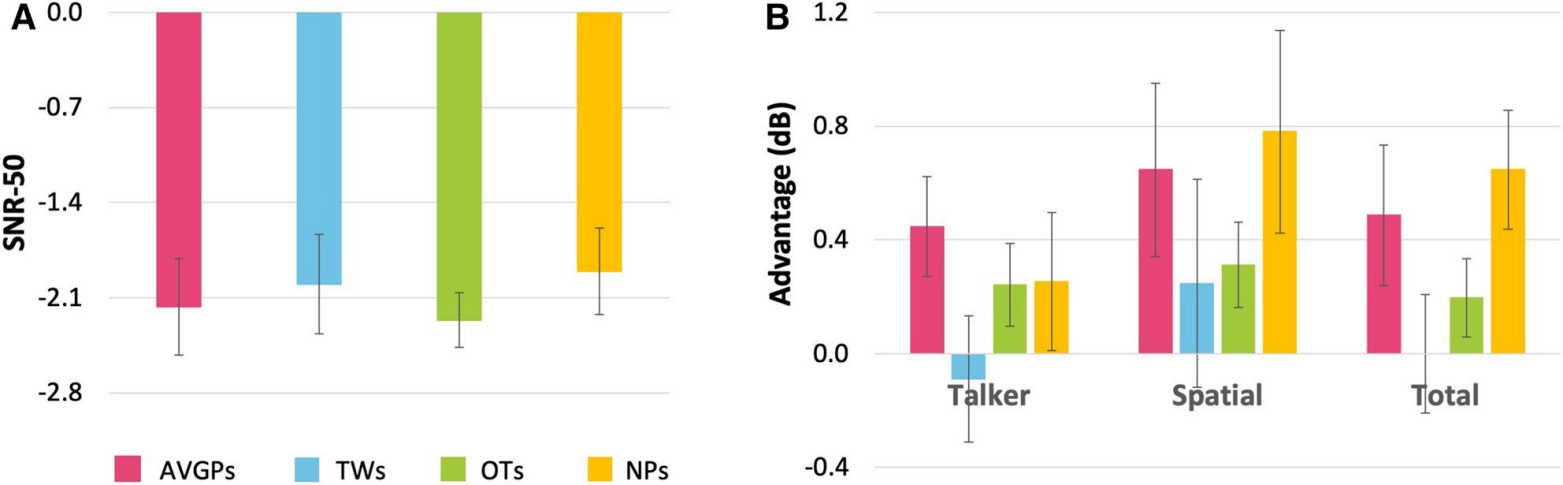
(**A**) Bamford–Kowal–Bench Speech-in-Noise (BKB-SiN): Auditory SiN task. A higher SNR-50 score would indicate a greater SNR necessary for successful verbal communication, a lower score indicates better listening in noise performance. (**B**) Listening in Spatialized Noise-Sentences (LiSN-S): auditory SiN task. A higher standardized score indicates better listening in noise performance. Pink: action video game players (AVGPs); blue: tweeners (TWs); green: others (OTs); yellow: non-players (NPs). Error bars show SEM.

The BKB-SiN presents simple sentences having 3–5 key words against a 4 talker ‘babble’ speech masker^34,35^. Performance is measured by the speech–noise ratio (SNR) required to attain 50% correct key word responses (SNR-50). All groups achieved similarly sensitive, low SNRs, indicating good listening in noise performance (Fig. 5A). A univariate ANOVA showed no significant differences between the groups (*p* = 0.80, η_p_^2^ = 0.013, BF_10_ = 0.10).

### Listening in Spatialized Noise-Sentences (LiSN-S)

Another test of speech hearing in noise, the LiSN-S^36,37^ measures ability to hear and recall spoken target sentences against a background of distracting talkers (Fig. 2C). The talkers may be the same or different voices, or come from the same or different directions. By subtracting performance on two versions of each condition, the LiSN-S achieves a degree of isolation between the auditory and cognitive contribution to each of three indices, Talker, Spatial and Total advantage^37^. All groups scored similarly on the standardized Talker, Spatial and Total Advantage scores, with higher scores indicating better performance (Fig. 5B). Univariate ANOVAs found no significant group differences (Talker: *p* = 0.34, η_p_^2^ = 0.044, BF_10_ = 0.23; Spatial: *p* = 0.48, η_p_^2^ = 0.033, BF_10_ = 0.17; Total: *p* = 0.12, η_p_^2^ = 0.075, BF_10_ = 0.54).

### Listening environments during play

**Figure 6:**
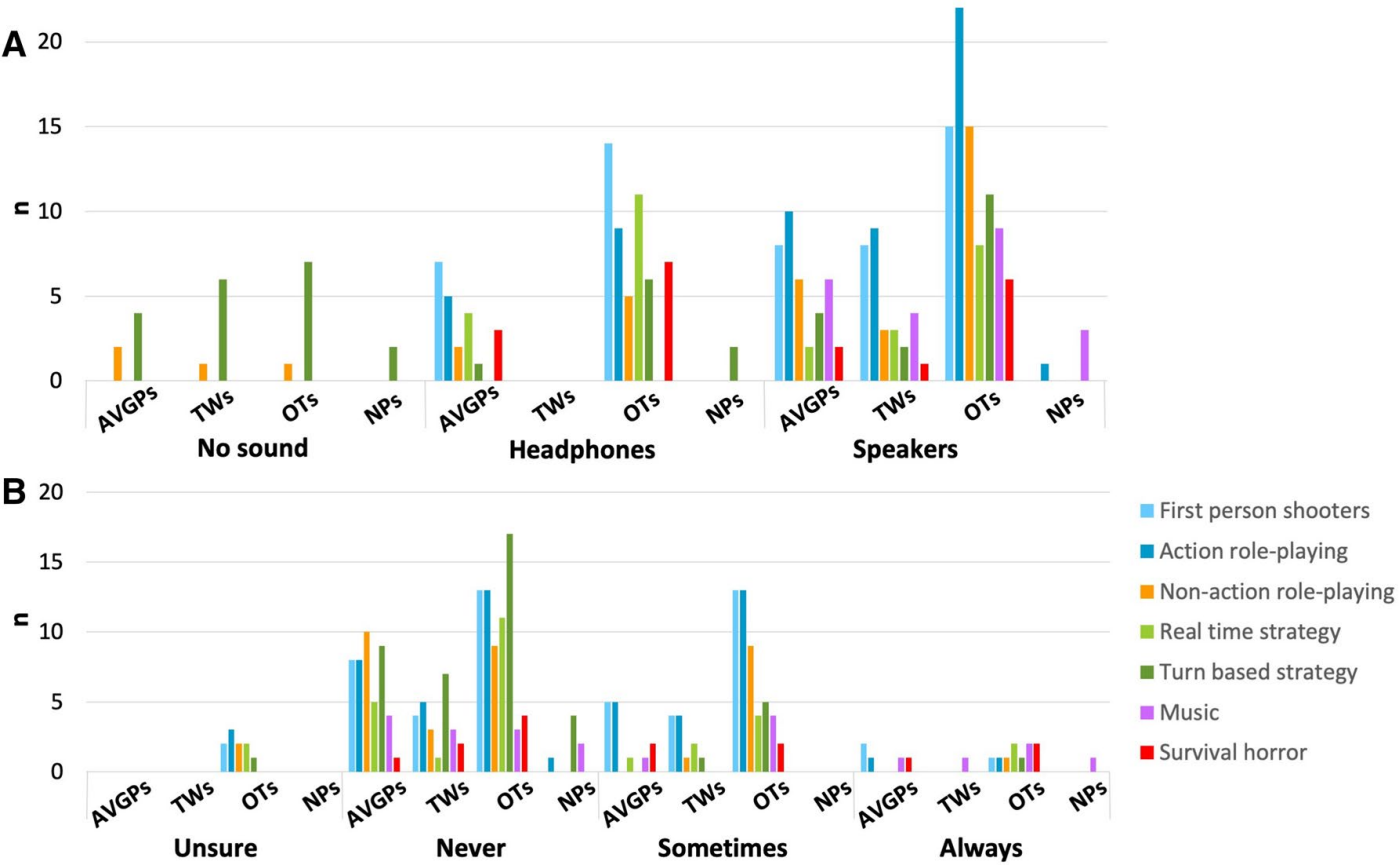
How participants who play video games listen to the audio of their computer games. (**A**) “How do you typically listen to these games?”. (**B**) “Do you play these games with surround sound? (e.g., Over a 5.1 speaker set up)”. Blue: action games; green: strategy games; orange: non-action role playing games; pink: music games; red: survival horror games.

As part of the background questionnaire completed during recruitment we collected data on how the participants played and interacted with their video games. During first person shooter games, 47% of AVGPS and 43% of OTs used headphones and 53% of AVGPs, 47% of OTs and 47% of TWs used open-field loudspeakers. In action role-playing games, 33% of AVGPs and 28% of OTs used headphones and 67% of AVGPs, 69% of OTs and 53% of TWs used loudspeakers (Fig. 6A). However, a range of listening environments was used. Only 13% of AVGPs and 3% of OTs used surround sound during first person shooters and 7% of AVGPs and 3% OTs during action role-playing games. Of those that responded, the majority (53% AVGPs, 41% OTs, 24–29% TWs) never used surround sound and about a third (33% AVGPs, 41% OTs, 24% TWs) only sometimes used the feature (Fig. 6B). Through discussions with participants, we found that while playing gamers (AVGPs, TWs and OTs) often simultaneously listened to a separate and irrelevant auditory source (e.g., television, podcasts).

## Discussion

This study assessed whether action video game play is associated with changes in visual and/or auditory skills. In terms of visual skills, pre-existing AVGPs were found to maintain better performance at higher set sizes as compared to NPs on the visual MOT task, replicating the highly cited finding that extensive action video game play is associated with enhanced visual cognition and perception^8^. However, we did not find any differences in performance on the auditory tasks between groups, suggesting that action video game play is not associated with a cross-modal benefit. Bavelier and colleagues (2012) proposed that all tasks AVGPs improve on may share the fundamental computational principle of making a decision based upon limited information in noise. However, we found this does not hold true for limited *auditory* information in noise, implying that ‘learning to learn’ has narrower boundaries than previously suggested and that probabilistic thinking is modality-specific. It may be that the probabilistic thinking process used to make judgements with limited *visual* information in noise is not adequate when dealing with the auditory system.

Green and colleagues (2010) found better performance by AVGPs on an auditory task compared to NPs. However, there are key differences in the function of the outcome measure used in that study compared to those used in the study reported here. First, TAiL is perceptually more demanding than the auditory tone location task used by Green. In TAiL, the task-relevant and -irrelevant information are part of the same sound object, while in Green’s task, the task-relevant (pure tone) and -irrelevant (white noise) information were separate sound objects. Second, the white noise used by Green et al. would have created energetic masking. The BKB-SiN and LiSN-S tasks used in this current study have speech babble maskers that create both informational and energetic masking^27,28^ thus generating ecologically-valid everyday listening environments.

Improvement in speech-in-noise ability has been found in a randomized, double-blind study^38^ showing that training on an audiomotor game leads to improved speech-in-noise ability in elderly hearing-impaired players, while training on an auditory working memory game does not. In Whitton’s study the training game involved interacting with auditory task-relevant stimuli where the participants monitored auditory feedback as they moved their finger through a virtual soundscape on a tablet device. The goal of the game was to complete a hidden puzzle by finding and rotating pieces. Participants improved their performance by monitoring the deviations between their expected and actual auditory feedback. Key to Whitton’s (2017) study, feedback was given through subtle variations in sound level, pitch or modulation rate, while ignoring task-irrelevant auditory information in the form of speech babble. It could be argued that, during play, participants were directly training *auditory* probabilistic thinking with task-relevant auditory stimuli. However, the task-irrelevant speech babble created informational and energetic masking mirroring the outcome measure (speech-in-noise). Therefore, using the definitions of Ferguson and Henshaw^4^, this training would be labeled as near transfer, while in the study reported here we were looking for evidence of far transfer training effects.

To be categorized as an AVGP in this study and previous studies e.g.,^8,30,39^ the participants had to be heavy players of action video games. This was defined as playing for, what some may consider, an extreme number of hours each week and over a prolonged period of time (see Table 1). Using these definitions we replicated the previous finding that AVGPs (n = 15) showed superior performance on the MOT task compared to NPs. However, we also extended our analysis into TWs (n = 17) and OTs (n = 32) to cover a wider range of action video game experience. Similar to the results of^30^ we found that, at least numerically, the TWs and OTs performed in between the AVGPs and NPs. This thus contributes to new directions in this field exploring the impact of groups beyond just the typical AVGP and NP populations that have been the focus of much of the literature thus far (e.g., to players of different genres or to intermediate players)^30,39,40^.

Musicians (professional and amateaur) have been found to have superior speech in noise perception compared to non-musicians e.g.,^41–43^; but see^44,45^. These musicians complete extensive auditory training by playing/writing/conducting music for many hours each week and over a prolonged time. Strait and Kraus^46^ suggest the interactive auditory environment musicians experience to be the key to their auditory learning. It may be that musicians are the auditory parallel to AVGPs in that musical training directly trains auditory probabilistic thinking in a similar way action video games trains visual probabilistic thinking.

The findings from our questionnaire on previous game play experience (Tables 1, 2; Fig. 1) suggest that a possible alternative reason for the absence of cross-modal benefit is that, for these gamers, interaction with the auditory information differed from interaction with the visual information in their gaming environment. This difference may stem from the fact that in the majority of video games visual information is task-relevant, while the auditory information is not. For example, visual information such as where the enemy is hiding or where an explosive was thrown is vital for successful game performance, whereas auditory information such as the sound of an explosion is considered an additional effect, rather than ‘life or death’ information within the game. Importantly, the auditory information usually lacks appropriate sound cues that, in this example, may include interaural differences indicative of the location of the explosion. Some games, for example those with surround sound, do make it possible to locate the source of the sound. However, the vast majority of our gamers reported that they did not have or listen to such meaningful sound.

A category of game where the auditory information is needed to successfully compete is audio games for the blind. In these ‘video-less’ games, images are replaced with musical cues and navigation by voice prompts, making audition the task-relevant modality. To maintain high performance, the auditory information in these games is vital. A game such as blind cricket (https://www.audiogamehub.com) could be used to assess the modality effects of game training by providing a controlled environment where the levels of visual and auditory information can be manipulated, for example by contrasting training using full visual graphics, reduced visual graphics (e.g., black and white with lower resolution), and only auditory cues. Such an investigation would provide a further assessment of whether probabilistic thinking requires modality-specific training.

### Limitations

This was a case–control study to assess the potential value of using action video games to train speech in noise abilities. Two limitations arise from the retrospective design of the study in terms of gaming experience. First, the participants’ auditory cognition and perception prior to their gaming experience were unknown, and therefore not controlled. Controlled intervention studies have found a causal relation between playing action video games and enhancement of visual cognition and perception e.g.,^9–12^. However, we were unable to assess whether prior auditory cognitive and perceptual ability affected auditory learning. Second, we used a questionnaire to gather data on the participants’ gaming history. Questionnaires have been shown to have a bias for both under and over-reporting prior behaviour; diaries and game play timers provide more accurate measurement e.g.,^47^. There is also evidence that the more games a participant plays the larger the discrepancies in their questionnaire responses^29^. However, questionnaires are able to reliably categorize the two ends of behavior^48,49^. In order to include a wide range of gaming experience in our analysis we expanded the categories investigated from the typical AVGP and NP to include TW (multi-genre game playing, mostly online) and OT (gaming experience not fitting a clear definition).

## Conclusion

While we replicate the finding that extensive action video game play is associated with better performance in the visual cognitive domain, we did not find a benefit for auditory cognition and perception. This suggests that the underlying probabilistic thinking video games are thought to improve may not be supramodal. If training probabilistic thinking is indeed modality-specific, then training using rich auditory information within a game format may lead to far transfer on auditory tasks. However, the acoustic characteristics of the game chosen may prove to be key.

## Methods

### Participants

A total of 85 individuals were recruited into this study through word of mouth and by using Institutional Review Board (IRB)-approved advertisements and materials via print, electronic, social and digital media at Cincinnati Children’s Hospital locations, and in the local and regional area. Five participants did not have normal hearing acuity (pure tone thresholds < 20 dB HL bilaterally at all octave frequencies from 250 to 8,000 Hz^50^ and did not go on for further testing. The remaining 80 participants’ ages ranged from 18–30 (M = 25.07 years, SD = 3.72 years, 29 females and 51 males) (Table 2). All procedures were approved by the IRB at Cincinnati Children’s Hospital Medical Center. At recruitment participants consented to completing screening questionnaires covering background information and gaming experience. Informed written consent was obtained from each participant prior to testing and they were compensated for their time and effort. All experiments were performed in accordance with relevant guidelines and regulations.

### Grouping

Participants were grouped into AVGPs, TWs, OTs and NPs by the number of hours spent playing different categories of games each week during the past year, and prior to the past year. The definitions of these groups can be found in Table 1, along with the break-down of gaming experience, regardless of categorization, in Fig. 1. We actively recruited participants that fitted the AVGP, TW and NP categories.

### Equipment

Participants were tested individually in a sound-attenuated booth. All tests were presented on a PC, with a 21 inch flat screen monitor placed in front of the participant at full screen brightness. All auditory stimuli were presented through Sennheiser 25 circumaural headphones. The MOT was presented using MATLAB v2016a. TAiL^51^ and LiSN-S^36^ were presented through their own, stand-alone software. The BKB-SiN task^52^ was played from its auditory recording. A horizontally placed custom made three choice button box was used to record reaction times in the MOT and TAiL. A hand print was placed in front of the button box to serve as a base for participants to place their dominant hand preceding each trial.

### Stimuli and procedure

Four tests were administered in a single testing session lasting approximately 2 h. The initial test was counterbalanced across participants using a Latin square design.

#### Visual Multiple Object Tracking (MOT)^17^

Each trial began with 16 dots moving in a random, continuous manner within a circular, grey background (Fig. 2A). Stimuli consisting of yellow and blue dots were presented for 10 s. Participants were instructed to focus on the central fixation cross. After 2 s, the blue dots turned yellow. Four seconds later one dot, the target stimulus, turned white and the participant was prompted to indicate the original color of the white dot. Participants continued to fixate on the cross throughout each trial while responding as quickly and accurately as possible. Participants each underwent one experimental block of 40 trials. Average RT and accuracy were calculated for each participant for 1 to 7 blue dots.

#### Test of Attention in Listening (TAiL)^33^

In each trial participants were presented with two 100 ms pure tones (gated on/off by 10 ms cos ramps) at 70 dB SPL with an inter-stimulus-interval of 300 ms (Fig. 2B). Tone pairs ranged from 476.2 to 6,187.5 Hz and were always at least 2.1 equivalent rectangular bandwidths (~ 4 semitones) apart. Using the button box, participants were tasked to indicate the correct response as quickly and accurately as possible. If the trial’s tones had the same task-relevant information (i.e. pitch in the attend-frequency condition; or ear presentation in the attend-location condition) participants were instructed to press the right button on the button box. If the trial’s tones differed in task-relevant information the participants were instructed to press the left button on the button box.

RT (from correct trials only) and accuracy were calculated for each TAiL condition for distraction and conflict resolution measures. Trials where the participant responded in less than 200 ms or longer than 2,500 ms were discarded in case of preemptive responding and interruption in performance. Distraction measures were calculated as the difference of responding to trials where the task-irrelevant information changed and trials where it did not, regardless of the task-relevant information (i.e. in the attend-location task: the difference between different frequency and same frequency trials, regardless of the location). Conflict resolution was calculated as the difference between incongruent and congruent trials (i.e. difference between trials where only one sound property changed and trials where both the sound properties changed or stayed constant).

Prior to testing, participants underwent practice trials for each condition in which they received a pass (60% correct) or fail. If passed, participants proceeded to do 3 blocks of 40 trials each for both conditions, a total of six blocks alternating between conditions. If failed, participants were given two more opportunities to complete the practice trials. If they still failed, they did not complete the blocks of TAiL testing.

#### Bamford–Kowal–Bench Speech-in-Noise (BKB-SiN)^35^

The BKB-SiN test is a standardized speech perception test utilizing a simultaneous four talker babble noise to simulate a realistic listening environment. The recording was presented with the babble noise at 65 dB SPL through binaural headphones with one sentence at each signal to noise ratio (SNR) ranging from + 21 to − 6 dB in 3 dB intervals. Participants repeated the target sentence with the tester marking their responses following standard BKB-SiN scoring.

#### Listening in Spatialized Noise-Sentences (LiSN-S)^36^

In this standardized test (Fig. 2C), a target signal was broadcast binaurally through headphones along with two other distracting signals. Both the target and distracting signals consisted of sentences spoken in American-English by an adult female. The target (T) and distractor (D1, D2) voices were manipulated with respect to talker (same voice, different voices) and direction (0°, ± 90° azimuth), creating four different listening conditions. From these four listening conditions, three difference scores were calculated: Talker advantage (different voices – same voice); Spatial advantage (different directions – same direction); and Total advantage (different voices and directions – same voices and directions).

Participants were asked to repeat the sentences of the target voice only. Distracting sentences remained constant at 55 db SPL. After each correct trial the target voice descended in level (4 dB), but if the participant incorrectly repeated back over 50% of the sentence the level increased (by 2 dB). The LISN-S software calculated the difference scores for each participant.

## Supplementary information

Supplementary Figure 1.

## Data Availability

The dataset generated during and analysed during the current study are available at GitHub: https://github.com/stewarthannahj/VGPs.git.
