# Supplementary material for: Auditory cognition and perception of action video game players
Source: Sci Rep. 2020 Sep 1;10:14410. doi: 10.1038/s41598-020-71235-z (PMC7462999; doi:10.1038/s41598-020-71235-z)

**Auditory cognition and perception of action video game players**

**Abbreviated title**: Video games and auditory training

Hannah J. Stewart ^1, 2^, Jasmin L. Martinez ^1, 3^, Audrey Perdew ^1^, C. Shawn Green ^4^ and

David R. Moore ^1, 5, 6^

1. Communication Sciences Research Center, Cincinnati Children’s Hospital Medical Center, Cincinnati, Ohio, USA
2. Division of Psychology and Language Sciences, University College London, London, UK
3. Department of Communication Sciences and Disorders, University of Cincinnati, Cincinnati, Ohio, USA
4. Department of Psychology, University of Wisconsin-Madison, Madison, Wisconsin, USA
5. Department of Otolaryngology, University of Cincinnati, Cincinnati, Ohio, USA
6. Manchester Centre for Audiology and Deafness, University of Manchester, UK

**Correspondence**: David R Moore, Ph.D.

Communication Sciences Research Center

Cincinnati Children’s Hospital Medical Center

Cincinnati, OHIO, 45229, USA

Email: david.moore2@cchmc.org

**Supplementary material**

Figure 1 shows the extrapolated weekly average hours played across the four gamer categories. It is important to note that such values should not be taken literally as they are from bins that were not equally sized. The middle of the bins were used in these calculations: 0 hours for ‘0 hours’; 0.5 hour for ‘0+ to 1 hour’; 2 hours for ‘1+ to 3 hours’; 4 hours for ‘3+ to 5 hours’; 7.5 hours for ‘5+ to 10 hours’; and 15 for ‘10+ hours’. It is possible that the average number of hours spent gaming are inflated or deflated.


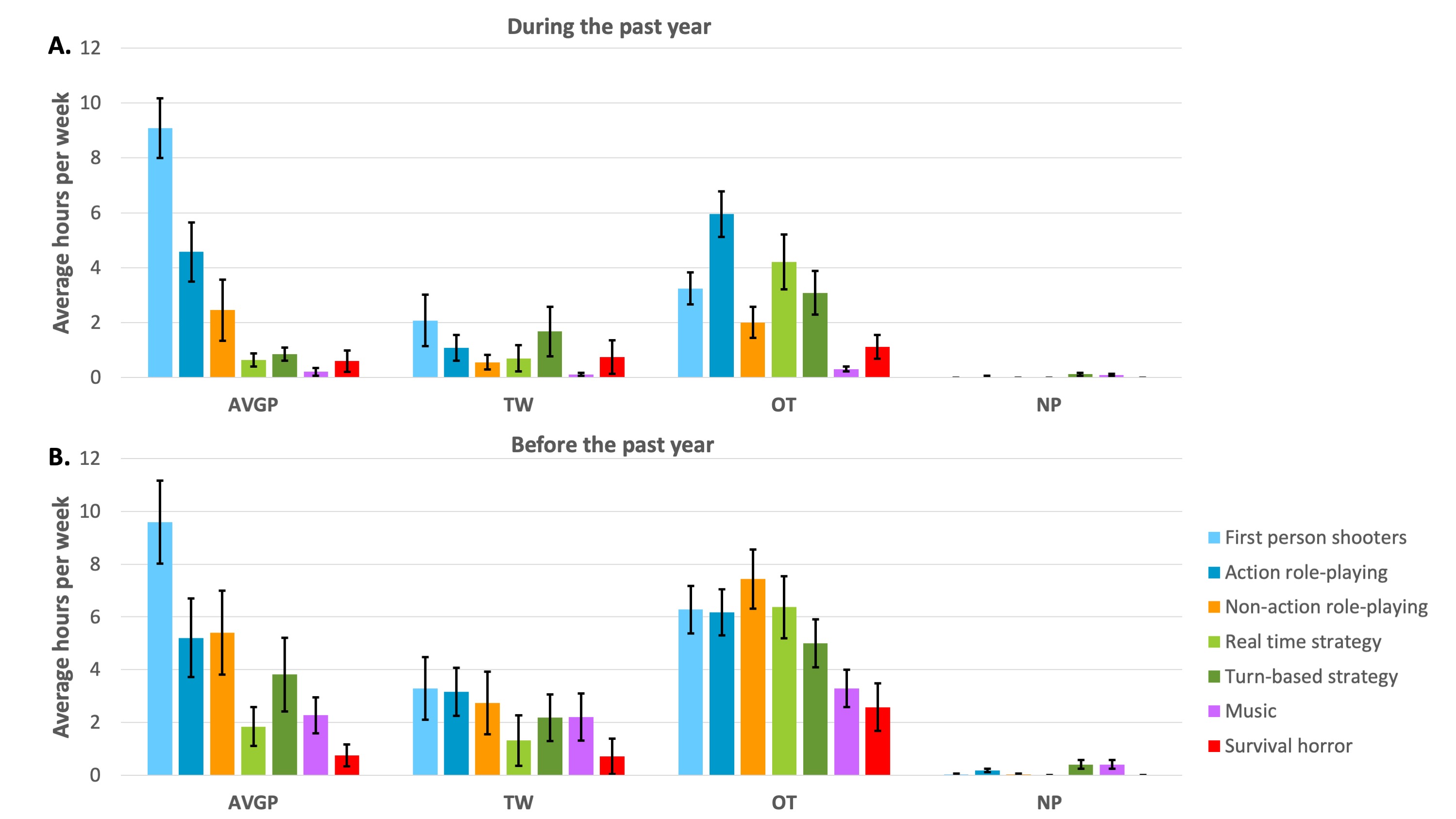

Supplement: Supplementary file 1 — Supplementary Figure 1. [file 41598_2020_71235_MOESM1_ESM.docx]
